# Optical coherence tomography tissue coverage and characterization at six months after implantation of bioresorbable scaffolds versus conventional everolimus eluting stents in the ISAR-Absorb MI trial

**DOI:** 10.1007/s10554-021-02251-x

**Published:** 2021-08-21

**Authors:** Himanshu Rai, Fernando Alfonso, Michael Maeng, Christian Bradaric, Jens Wiebe, Javier Cuesta, Evald Høj Christiansen, Salvatore Cassese, Petra Hoppmann, Roisin Colleran, Fiona Harzer, Jola Bresha, Nejva Nano, Simon Schneider, Karl-Ludwig Laugwitz, Michael Joner, Adnan Kastrati, Robert A. Byrne

**Affiliations:** 1 Deutsches Herzzentrum München, Technische Universität München, Munich, Germany; 2 Hospital Universitario de La Princesa Madrid, Madrid, Spain; 3 Aarhus University Hospital, Aarhus, Denmark; 4 Medizinische Klinik Und Poliklinik Innere Medizin I, Klinikum Rechts Der Isar, Technische Universität München, Munich, Germany; 5 DZHK (German Centre for Cardiovascular Research), Partner Site Munich Heart Alliance, Munich, Germany; 6 Cardiovascular Research Institute Dublin, Mater Private Network, Dublin, Ireland; 7 School of Pharmacy and Biomolecular Sciences, RCSI University of Medicine and Health Sciences, Dublin, Ireland

**Keywords:** Bioresorbable scaffold, Acute myocardial infarction, Malapposition, Optical coherence tomography, Grey-scale signal intensity, Uncovered struts

## Abstract

**Purpose:**

Data regarding vessel healing by optical coherence tomography (OCT) after everolimus-eluting bioresorbable scaffolds (BRS) or everolimus-eluting metallic stent (EES) implantation in acute myocardial infarction (AMI) patients is scarce. We compared OCT findings after BRS or EES implantation in patients with AMI enrolled in a randomized trial.

**Methods:**

In ISAR-Absorb MI, AMI patients were randomized to BRS or EES implantation, with 6–8 month angiographic follow-up. This analysis includes patients who underwent OCT during surveillance angiography. Tissue characterization was done using grey-scale signal intensity analysis. The association between OCT findings and target lesion failure (TLF) at 2 years was investigated.

**Results:**

OCT was analyzed in 103 patients (2237 frames, 19,827 struts) at a median of 216 days post-implantation. Of these, 70 were treated with BRS versus 32 with EES. Pre-(92.8 vs. 68.7%, p = 0.002) and post-dilation (51.4 vs. 12.5%, p < 0.001) were more common in BRS as compared to EES. Strut coverage was higher in BRS vs. EES (97.5% vs. 90.9%, p < 0.001). Mean neointimal thickness was comparable in both groups [85.5 (61.9, 124.1) vs. 69.5 (32.7, 127.5) µm, respectively, p = 0.20]. Mature neointimal regions were numerically more common in BRS (43.0% vs. 24.6%; p = 0.35); this difference was statistically significant in ST-elevation myocardial infarction patients (40.9% vs. 21.1%, p = 0.03).

At two-years, 8 (7.8%) patients experienced TLF. Mean neointimal area [0.61 (0.21, 1.33) vs. 0.41 (0.11, 0.75) mm^2^, p = 0.03] and mean neointimal coverage [106.1 (65.2, 214.8) vs. 80.5 (53.5, 122.1) µm, p < 0.01] were higher, with comparable tissue maturity, in lesions with versus without TLF.

**Conclusions:**

In selected patients who underwent OCT surveillance 6–8 months after coronary intervention for AMI with differing implantation characteristics depending on the device type used, vessel healing was more advanced in BRS compared with EES, particularly in the STEMI subgroup.

**Supplementary Information:**

The online version contains supplementary material available at 10.1007/s10554-021-02251-x.

## Introduction

Everolimus-eluting bioresorbable scaffolds (BRS) were designed with the intention of overcoming the long-term limitations of conventional metallic drug eluting stents (DES) [1]. The technology aims to provide temporary mechanical scaffolding with anti-proliferative drug release in the early period after implantation, with slow resorption thereafter to eliminate any nidus for late stent failure as seen with DES, caused by restenosis or stent thrombosis. It was hypothesized that complete resorption would facilitate return of vasomotor function and expansive remodeling of the treated arterial segment late after implantation.

Randomized clinical trials have shown significantly higher rates of target lesion failure and device thrombosis with the everolimus-eluting BRS as compared to conventional stents at mid- and long-term follow-up [2, 3], However, one area where BRS may offer an advantage over metallic stents is the setting of ST-elevation myocardial infarction (STEMI). Two trials have compared BRS and conventional EES exclusively in patients with acute myocardial infarction (AMI) [4, 5] The ISAR-Absorb MI trial showed comparable angiographic outcomes after BRS- or EES-implantation at 6–8 months. The ABSORB STEMI-TROFI II trial showed a lower healing score (as assessed by OCT) within BRS compared with EES at 6 months. Although neither trial was adequately powered to assess clinical outcomes, a meta-analysis of individual patient data from these two trials showed comparable clinical outcomes in the BRS and EES groups at follow-up, although this analysis was also impacted by limited power [2].

The present study involves a subset of subjects enrolled in the ISAR-Absorb MI trial who underwent optical coherence tomography (OCT) surveillance at the time of angiographic follow-up. Our primary objective was to investigate differences in the vessel healing processes 6–8 months after BRS- versus EES-implantation as assessed by OCT. Our secondary objective was to identify OCT factors associated with subsequent target lesion failure (TLF) out to 2 years post-stenting.

## Materials and methods

### Patient population

This study includes the subset of patients enrolled in the **I**ntracoronary **S**caffold **A**ssessment a **R**andomized evaluation of **Absorb** in **M**yocardial **I**nfarction (**ISAR-Absorb MI**) Trial who underwent OCT surveillance at the time of routine angiographic follow-up at 6–8 months. OCT surveillance at the time of angiographic follow-up was not protocol-mandated and was done at the operator’s discretion. Subjects who underwent target lesion revascularization before or at 6–8 month angiographic follow-up were excluded.

ISAR-Absorb MI was an investigator-initiated, prospective, randomized, multicenter, non-inferiority, clinical trial with a 2:1 treatment allocation to everolimus-eluting BRS (Absorb; Abbott Vascular, Santa Clara, CA, USA) vs. durable polymer everolimus-eluting stents (EES) in patients undergoing percutaneous coronary intervention for AMI (ClinicalTrials.gov Identifier: NCT01942070). Details of the trial design and primary results have been published elsewhere [5]. In brief, patients > 18 years presenting with STEMI or NSTEMI, if accompanied by visual evidence of thrombosis on angiography, with planned stent implantation in a de novo lesion in a native vessel or coronary bypass graft with a reference vessel diameter of ≥ 2.5 mm and ≤ 3.9 mm were included. Key exclusion criteria included target lesions located in the left main coronary artery, severely calcified lesions, bifurcation lesions with a side branch diameter > 2 mm, and any comorbid conditions with a life expectancy < 1 year or that might result in protocol non-compliance. OCT surveillance at 6–8 month angiographic follow-up was done at the investigator’s discretion. At three of the five participating centers, OCT surveillance was done routinely as part of clinical practice. All images obtained were sent to a centralized core laboratory [Intracoronary Stenting and Antithrombotic Research (ISAR) Center, Deutsches Herzzentrum München, Munich, Germany] as raw data for off-line analyses. Angiographic sequences and OCT pullbacks were measured by independent readers experienced in quantitative coronary angiography (QCA) and OCT analysis. Patients underwent additional clinical follow-up at two years post-procedure (16–18 months after angiographic/OCT surveillance) during which TLF incidence was recorded. TLF was defined as a composite of cardiac death, target-vessel myocardial infarction and target lesion revascularization.

### QCA analysis

QAngio XA 7.3.96.0 (Medis Medical Imaging Systems, Leiden, NL) was used for QCA analysis. Measurements were performed on cineangiograms recorded after the administration of intracoronary nitro-glycerine using the same single worst-view projection at all times. The contrast-filled non-tapered catheter tip was used for calibration before quantitative analysis. Both “in-stent” (stented segment) and “in-segment” (5-mm margins proximal and distal to the stent) areas were analyzed. Late lumen loss was defined as the difference between the minimal luminal diameter at the end of the procedure and the minimal luminal diameter at follow-up angiography. Binary angiographic restenosis was defined as diameter stenosis of > 50% in the in-segment area at follow-up. Standard criteria were used for determining qualitative morphological lesion characteristics.

### OCT analysis

OCT pullbacks were acquired using a standard non-occlusive technique with a Frequency Domain-OCT intravascular imaging system (Abbott Laboratories, Illinois, USA) and a Dragonfly™ DUO™ or OPTIS™ catheter (Abbott Laboratories, Illinois, USA). Unitary image acquisition length was 75 mm or 54 mm, while the pullback acquisition rate was 36 or 18 mm/sec respectively.

QIvus 3.0.30.0 software (Medis Medical Imaging Systems, Leiden NL) was used to perform morphometric analysis as per standard operating procedure of the OCT core-lab. Contiguous cross-sections within the stented segment spaced at each 1 mm longitudinal interval were analyzed. Parameters assessed for morphometric analysis included stent area, lumen area, stent diameter, neointimal area, neointimal thickness and percentage area of stenosis [6]. Stent/scaffold expansion index was calculated according to the criteria using the formula: minimum stent or scaffold area/reference lumen area [7, 8]. Struts were adjudicated as covered when the neointimal tissue overlying each stent strut was ≥ 20 µm (minimum axial resolution of OCT) and uncovered if the neointimal tissue overlying each strut was < 20 or < 30 μm (for EES and BRS, respectively) [9, 10]. We assumed some degree of irregular degradation of poly-d,l-lactide coating along with the poly-l-lactic acid strut backbone at 6–8 month post-implant follow-up. Accordingly, we regarded the minimum distance from the strut surface to the lumen contour (in the direction of the gravitational center of the vessel) as neointimal thickness overlying that strut. Struts were adjudicated as malapposed when strut center-to-lumen contour distance was more than: stent/scaffold strut thickness + polymer thickness + minimal axial resolution of OCT. Struts located at the ostium of side branches, with no vessel wall behind, were designated as non-apposed side-branch (NASB) struts and were excluded from the apposition analysis.

### GSI analysis

Neointimal tissue characterization as mature or immature was performed on OCT cross sections using offline GSI analysis. OCT images were manually transferred to an image editing software (ImageJ, Version 1.48 g, 2013) at a resolution of 1024 × 1024 pixel and converted to a grey scale signal (8-bit) as preparation for GSI analysis. Contiguous cross-sections within the stented segment spaced at 1 mm longitudinal intervals were analyzed. The neointimal region of interest (ROI) above each covered stent strut was delineated and 256-level GSI was measured for every pixel within the ROI. GSI analysis was performed only for neointimal ROIs with a thickness of 100 to 400 µm. Tissue coverage was classified as mature or immature according to a standard cut-off GSI score of 109.7 based on a prior pre-clinical study and a pilot clinical investigation [11].

### Statistical analysis

Continuous data are presented as mean ± SD or median (Interquartile range, IQR). Categorical data are presented as observed frequencies and proportions (%). Morphometric OCT parameters and GSI results were compared between the two stent groups (BRS vs. EES) for the whole cohort and for the STEMI subgroup. Categorical variables were compared between the two stent groups using the chi^2^ test with Yates’ continuity correction or the Fisher’s exact test (where at least one expected cell value was expected to be < 5). Continuous variables were compared using Student’s t-test or Wilcoxon rank sum test, as appropriate. Generalized linear mixed models (GLMM) were used as appropriate to account for cluster variability. Inter-and intra-observer variability for strut coverage, apposition and GSI-aided tissue characterization was evaluated using Cohen’s Kappa (κ) coefficient in ~ 5% of frames, which were randomly selected. A two-sided *P-value* ≤ 0.05 was considered as an indicator of statistical significance. All statistical analyses were performed using R (version 3.5.0, R Core Team, R Foundation, Vienna, Austria).

## Results

**Fig. 1:**
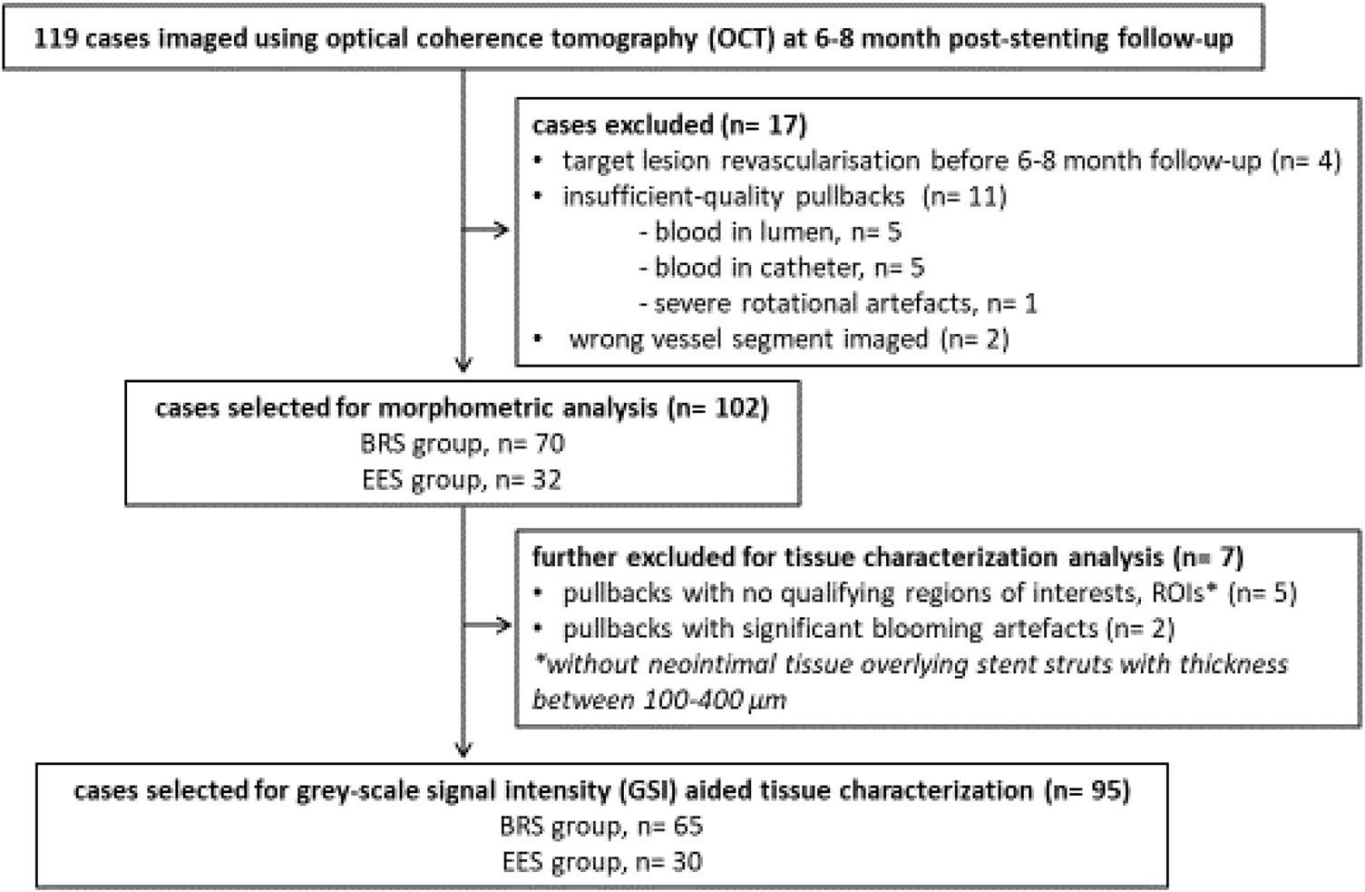
Study flow chart

A total of 119 cases enrolled in ISAR-Absorb-MI had OCT imaging performed at the time of 6–8 month angiographic follow-up. Of these, 17 failed the selection process for morphometric analysis. The remaining 102 cases were suitable for morphometric analysis (70 BRS cases; 32 EES cases); of these 95 cases were suitable for GSI analysis (65 BRS cases; 30 EES cases) (Fig. 1).

There were differences observed between the baseline and procedural characteristics of patients with and without OCT follow-up suitable for morphometric analysis (see Supplementary Table 1 and Supplementary Table 2).

**Table 1:** Baseline patient characteristics

	BRS	EES	P-value
*Patients*	70	32	
Age, years	59.3 ± 9.5	64.3 ± 11.2	0.03
Female gender	7 (10.0)	7 (21.9)	0.11
Diabetes mellitus	14 (20.0)	5 (15.6)	0.60
Insulin dependent	2 (2.9)	0 (0)	0.33
Hypertension	31 (44.3)	13 (40.6)	0.73
Current smoking	37 (52.9)	17 (53.1)	0.98
Family history of CAD	10 (14.3)	2 (6.2)	0.30
Prior percutaneous coronary intervention	4 (5.7)	1 (3.1)	0.67
Prior myocardial infarction	4 (5.7)	0 (0)	0.17
Number of vessels diseased			0.15
1 Vessel disease	53 (75.7)	19 (59.4)	
2 Vessel disease	12 (17.1)	7 (21.9)	
3 Vessel disease	5 (7.1)	6 (18.7)	
Clinical presentation			0.19
ST-elevation myocardial infarction	60 (85.7)	24 (75.0)	
Non-ST-elevation myocardial infarction	10 (14.3)	8 (25.0)	
STEMI location/presentation			0.14
Anterior	31 (51.7)	10 (41.7)	
Lateral	9 (15.0)	1 (4.2)	
Posterior	20 (33.3)	13 (54.2)	
Troponin (max), ng/dl	6.09 ± 9.56	4.44 ± 4.65	0.25

Data shown as mean ± SD or number (percentage)

**Table 2:** Baseline lesion and angiographic characteristics

	BRS	EES	P-value
*Lesions*	70	32	
Target vessel			0.18
Left anterior descending	34 (48.6)	14 (43.7)	
Left circumflex	12 (17.1)	2 (6.2)	
Right coronary artery	24 (34.3)	16 (50.0)	
Bifurcation	15 (21.7)	6 (18.7)	0.73
Pre-dilation	64 (92.8)	22 (68.7)	0.002
Stent diameter, max (mm)	3.1 ± 0.4	3.1 ± 0.4	0.96
Total stented length (mm)	21.4 ± 9.6	22.8 ± 10.7	0.54
Nominal diameter of largest balloon (mm)	3.2 ± 0.4	3.2 ± 0.4	0.51
Balloon pressure, max (atm)	17.7 ± 3.2	16.6 ± 3.1	0.10
Post-dilation	36 (51.4)	4 (12.5)	< 0.001
TIMI flow, post PCI			0.28
0	0 (0)	1 (3.1)	
1	0 (0)	0 (0)	
2	1 (1.4)	1 (3.1)	
3	69 (98.6)	30 (93.7)	
*Quantitative coronary angiography analysis*			
Pre-intervention			
Reference diameter (mm)	2.85 ± 0.39	2.88 ± 0.37	0.71
Minimal lumen diameter (mm)	0.30 ± 0.36	0.16 ± 0.34	0.06
Diameter stenosis (%)	89.6 ± 12.7	94.8 ± 11.2	0.04
Post-intervention			
Reference diameter (mm)	2.95 ± 0.39	2.98 ± 0.37	0.65
Minimal lumen diameter, in-stent (mm)	2.59 ± 0.36	2.69 ± 0.37	0.24
Minimal lumen diameter, in-segment (mm)	2.31 ± 0.45	2.26 ± 0.44	0.66
Diameter stenosis, in-stent (%)	11.8 ± 5.9	9.9 ± 5.6	0.13
Diameter stenosis, in-segment (%)	22.0 ± 9.3	24.1 ± 12.7	0.41

Data shown as mean ± SD or number (percentage)

Baseline patient characteristics are shown in Table 1. Patients in the BRS group as compared to EES group were younger (59.3 ± 9.5 vs. 64.3 ± 11.2 years, respectively, p = 0.03) but the groups were otherwise well matched. Lesion and procedural characteristics are shown in Table 2. Pre-dilation (92.8% vs. 68.7%, p = 0.002) and post-dilation (51.4% vs. 12.5%, p < 0.001) were more frequently done in patients treated with BRS. Lesion and procedural characteristics were otherwise well matched between the two groups.

**Fig. 2:**
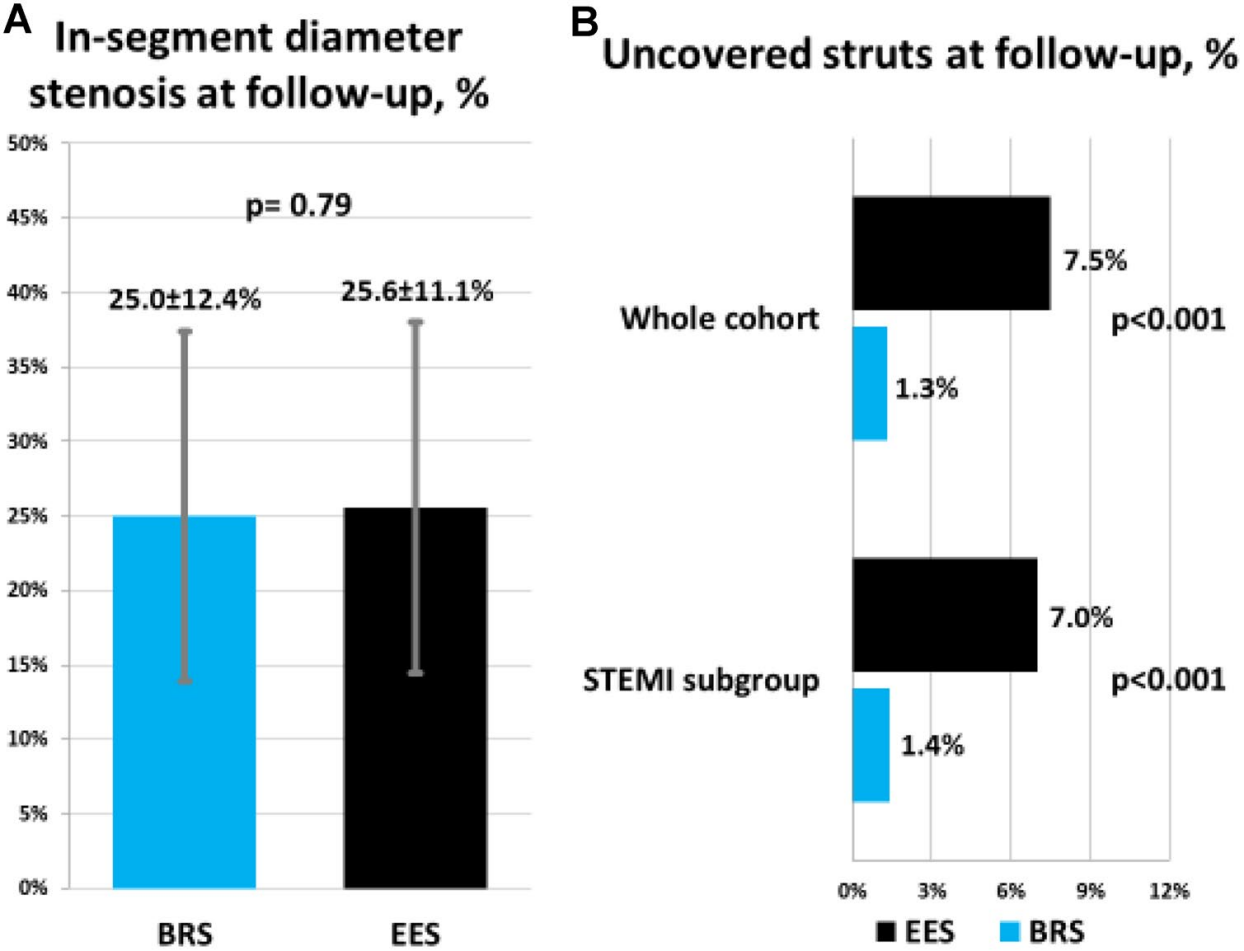
Principal angiographic and OCT findings at 6–8-month follow-up. **a** Comparison of in-segment percentage diameter stenosis between BRS and EES; **b** Proportion of uncovered struts at 6–8 months follow-up between BRS and EES

Angiographic and OCT surveillance of patients in the present study was performed at a median follow-up was 216 days post-intervention. QCA results of follow-up angiogram are shown in Table 3. In-segment diameter stenosis, which was the primary endpoint measure in the main study, was comparable between BRS and EES (25.0 ± 12.4 vs. 25.6 ± 11.1%, p = 0.79). (Fig. 2, Panel A) In-stent and in-segment minimal lumen diameters and late lumen loss were also comparable between the two treatment groups.

**Table 3:** Angiographic follow-up at 6–8 months

	BRS	EES	P-value
*Lesions*	70	32	
Days to angiographic follow-up	216 [204, 233]	215 [205, 239]	0.70
Reference diameter, mm	2.90 ± 0.43	2.97 ± 0.38	0.46
Minimal lumen diameter, in-stent (mm)	2.38 ± 0.47	2.51 ± 0.53	0.23
Minimal lumen diameter, in-segment (mm)	2.18 ± 0.50	2.21 ± 0.45	0.78
Diameter stenosis, in-stent (%)	18.0 ± 11.6	15.7 ± 12.3	0.39
Diameter stenosis, in-segment (%)	25.0 ± 12.4	25.6 ± 11.1	0.79
Late lumen loss, in-stent (mm)	0.21 ± 0.29	0.17 ± 0.36	0.60
Late lumen loss, in-segment (mm)	0.12 ± 0.38	0.05 ± 0.45	0.47
Binary restenosis	5 (7.1)	1 (3.1)	0.42

Data shown as mean ± SD or median [IQR] or number (percentage)

**Fig. 3:**
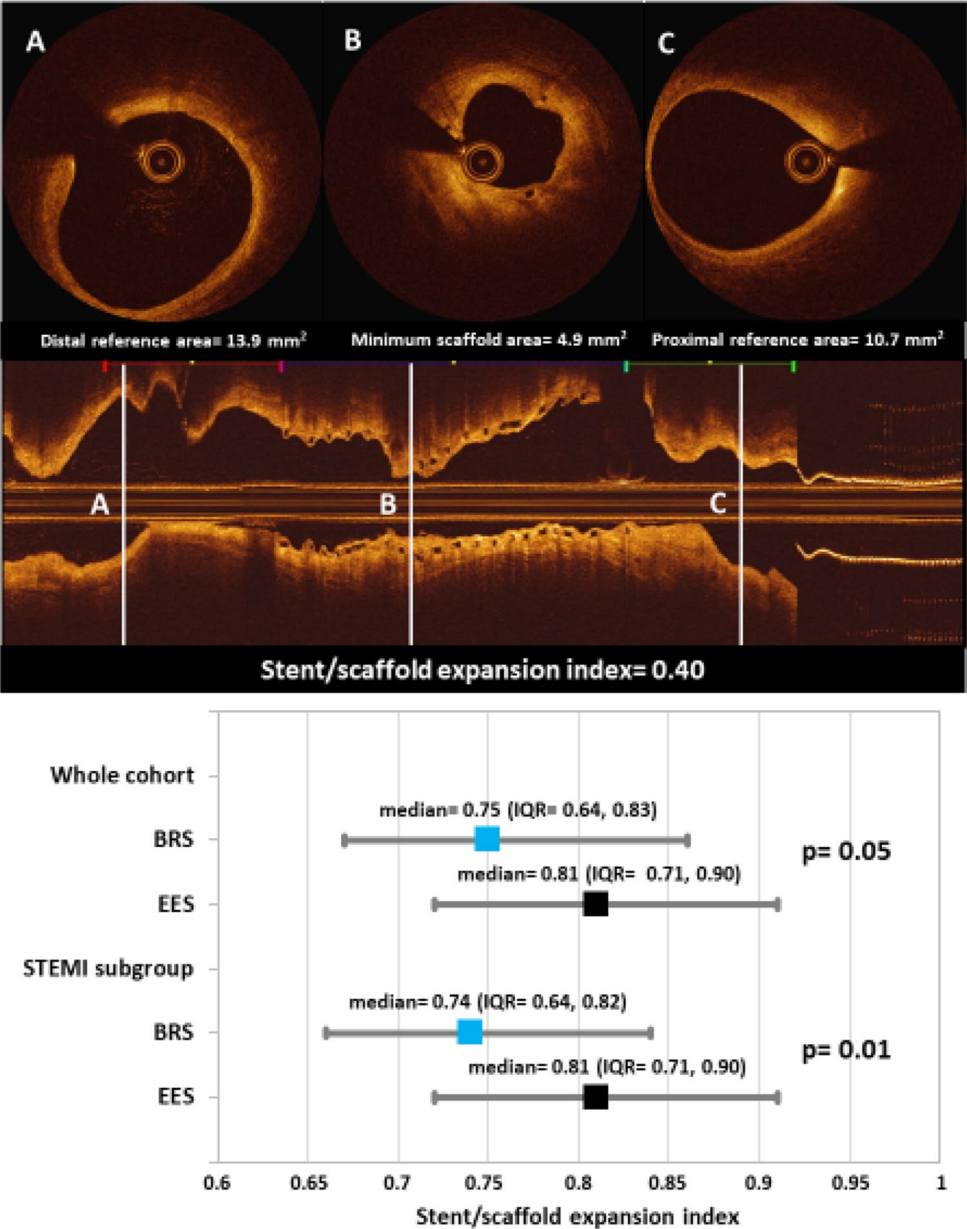
Comparison of stent/scaffold expansion indices between BRS and EES

OCT analysis results are shown in Table 4. In total, 2237 frames with 19,827 struts were assessed. Minimum lumen area [5.13 (3.95, 6.70) vs. 4.93 (3.84, 6.99) mm^2^] and minimum stent area [5.78 (4.88, 7.34) vs. 6.39 (4.77, 7.45) mm^2^] were comparable between BRS and EES in the whole cohort, as well as in the STEMI subgroup. Stent/scaffold expansion index was marginally lower in BRS as compared to EES in the whole cohort [0.75 (0.64, 0.83) vs. 0.81 (0.71, 0.90), p = 0.05], and significantly lower in BRS in the STEMI subgroup [0.74 (0.64, 0.82) vs. 0.81 (0.71, 0.90), p = 0.01] (Fig. 3).

**Table 4:** Results from morphometric OCT analysis

	Whole cohort			STEMI subgroup		
	BRS	EES	*P-value*	BRS	EES	*P-value*
Patient-level measurements						
*Patients, n*	70	32		60	24	
Stented length, mm	19.8 (13.5, 24.5)	21.7 (16.6, 26.6)	0.77	19.25 (13.47, 23.85)	21.7 (16.92, 27.5)	0.53
Reference lumen diameter, mm	3.25 (2.81, 3.70)	3.11 (2.73, 3.46)	0.31	3.3 (2.83, 3.77)	2.98 (2.73, 3.36)	0.09
Reference lumen area, mm^2^	8.38 (6.33, 10.88)	7.65 (5.80, 9.44)	0.22	8.60 (6.35, 11.19)	7.02 (5.80, 8.86)	0.04
Minimum lumen diameter, mm	2.56 (2.24, 2.92)	2.50 (2.21, 2.98)	0.97	2.53 (2.24, 2.85)	2.47 (2.21, 2.82)	0.58
Maximum lumen diameter, mm	3.26 (2.92, 3.86)	3.28 (2.87, 3.75)	0.64	3.22 (2.92, 3.80)	3.2 (2.87, 3.73)	0.40
Minimum lumen area, mm^2^	5.13 (3.95, 6.70)	4.93 (3.84, 6.99)	0.96	5.04 (3.94, 6.84)	4.79 (3.84, 6.26)	0.49
Maximum lumen area, mm^2^	8.35 (6.70, 11.71)	8.49 (6.46, 11.03)	0.59	8.17 (6.67, 11.34)	8.05 (6.46, 10.95)	0.32
Minimum stent diameter, mm	2.71 (2.47, 3.06)	2.88 (2.46, 3.09)	0.52	2.69 (2.46, 3.14)	2.88 (2.46, 3.09)	0.65
Maximum stent diameter, mm	3.29 (2.90, 3.78)	3.29 (2.89, 3.59)	0.40	3.27 (2.89, 3.67)	3.26 (2.89, 3.59)	0.50
Minimum stent area, mm^2^	5.78 (4.88, 7.34)	6.39 (4.77, 7.45)	0.73	5.70 (4.83, 7.72)	6.19 (4.77, 7.32)	0.95
Maximum stent area, mm^2^	8.52 (6.67, 11.25)	8.50 (6.53, 10.14)	0.30	8.41 (6.64, 10.57)	8.36 (6.53, 10.14)	0.37
Minimum neointimal area, mm^2^	-0.41 (-1.02, -0.01)	-0.23 (-0.63, 0.09)	0.78	-0.415 (-1.05, 0.01)	-0.20 (-0.63, 0.14)	0.24
Maximum neointimal area, mm^2^	1.09 (0.71, 1.75)	1.30 (0.73, 1.57)	0.49	1.01 (0.70, 1.49)	1.35 (0.98, 1.70)	0.16
Stent/scaffold expansion index	0.75 (0.64, 0.83)	0.81 (0.71, 0.90)	0.05	0.74 (0.64, 0.82)	0.81 (0.71, 0.90)	0.01
Frame-level measurements						
Assessed frames, n	1,529	708		1,268	528	
Lumen diameter, mm	2.91 (2.58, 3.34)	2.94 (2.56, 3.31)	0.84	2.88 (2.58, 3.35)	2.83 (2.54, 3.23)	0.61
Lumen area, mm^2^	6.64 (5.23, 8.74)	6.79 (5.15, 8.63)	0.76	6.52 (5.22, 8.83)	6.30 (5.07, 8.21)	0.53
Stent diameter, mm	3.02 (2.69, 3.42)	3.10 (2.70, 3.40)	0.89	2.99 (2.68, 3.42)	3.04 (2.67, 3.36)	0.83
Stent area, mm^2^	7.13 (5.69, 9.16)	7.57 (5.73, 9.06)	0.83	7.00 (5.64, 9.19)	7.26 (5.61, 8.86)	0.77
Neointimal area, mm^2^	0.41 (0.10, 0.77)	0.43 (0.15, 0.77)	0.73	0.40 (0.09, 0.72)	0.49 (0.19, 0.86)	0.17
Percentage area stenosis, %	6.15 (1.27, 11.22)	5.95 (2.06, 12.16)	0.77	5.75 (1.18, 10.84)	7.43 (2.43, 13.13)	0.20
Strut-level measurements						
*Visible struts, n*	12,704	7,123		10,527	5,236	
Covered struts, %	97.5	90.9	< 0.001	97.4	91.5	< 0.001
Uncovered struts, %	1.3	7.5	< 0.001	1.4	7.0	< 0.001
Malapposed struts, %	0.5	1.1	0.51	0.5	0.9	0.58
Non-apposed side branch struts, %	0.7	0.5	0.56	0.7	0.6	0.61
Neointimal coverage, µm	85.5 (61.9, 124.1)	69.5 (32.7, 127.5)	0.20	82.7 (60.5, 118.8)	76.6 (36.2, 139.5)	0.84

Data shown as numbers, percentages or median (IQR)

Amongst frame level measurements, neointimal area [0.41 (0.10, 0.77) vs. 0.43 (0.15, 0.77) mm^2^, p = 0.73] and percentage area stenosis [6.15 (1.27, 11.22) vs. 5.95 (2.06, 12.16) %, p = 0.77] as well as rest of the parameters were comparable between BRS and EES in the whole cohort.

The percentage of uncovered struts was significantly less common in BRS as compared to EES in the whole cohort (1.3% vs. 7.5%, p < 0.001), as well as in the STEMI subgroup (1.4% vs. 7.0%, p < 0.001) (Fig. 2, Panel B). Amongst other strut-level measurements, strut coverage was found to better with BRS compared to EES (97.5% vs. 90.9%, p < 0.001). Malapposed struts were numerically less common with BRS (0.5% vs. 1.1%, p = 0.51). Neointimal thickness was also numerically higher in BRS as compared to EES [69.5 (32.7, 127.5) vs. 85.5 (61.9, 124.1) µm, p = 0.20]. Results were consistent for each parameter in the STEMI subgroup. Inter-and intra-observer variability for strut apposition assessed randomly in 122 frames (1072 struts) showed high concordance (κ = 0.91 and κ = 0.93, respectively).

**Fig. 4:**
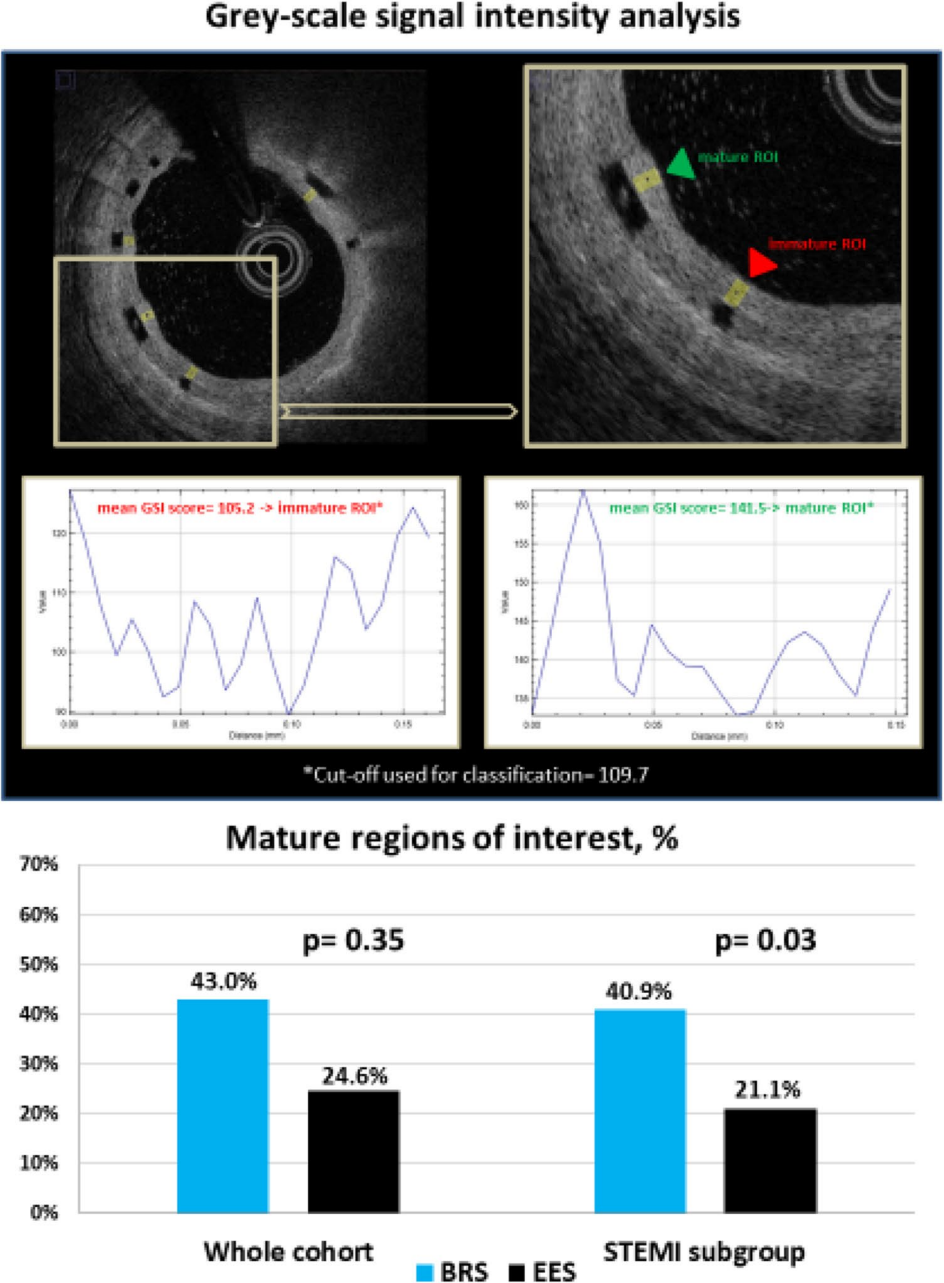
Proportion of mature regions of interest in BRS and EES. Cut-off GSI score used for classification = 109.7 (Malle et al., Arteriosclerosis, thrombosis, and vascular biology. 2013;33(6):1376–83.) [11]

Results of tissue characterization by GSI analysis are summarized in Table 5. Mature ROIs were numerically more common in BRS compared to EES (43.0% vs. 24.6%; p = 0.35); this difference was statistically significant in STEMI subgroup (40.9% vs. 21.1%, p = 0.03) (Fig. 4). Inter-and intra-observer variability for neointimal tissue characterization assessed in 117 frames (219 ROIs) also showed high concordance (κ = 0.90 and κ = 0.90, respectively).

**Table 5:** Tissue characterization by grey-scale signal intensity analysis

	Whole cohort			STEMI subgroup		
	BRS	EES	*P-value*	BRS	EES	*P-value*
Patients, n	65	30		55	22	
Frames analyzed, n	728	360		558	276	
Regions of interest analyzed, n	2,233	1,210		1,601	954	
Mean grey-scale signal intensity score	105.8 (91.0, 121.0)	95.9 (78.7, 109.6)	0.29	104.8 (90.6, 119.4)	92.0 (74.0, 107.4)	0.02
Mature regions of interest, %	43.0	24.6	0.35	40.9	21.1	0.03

Data shown as numbers, percentages or median (IQR)

**Fig. 5:**
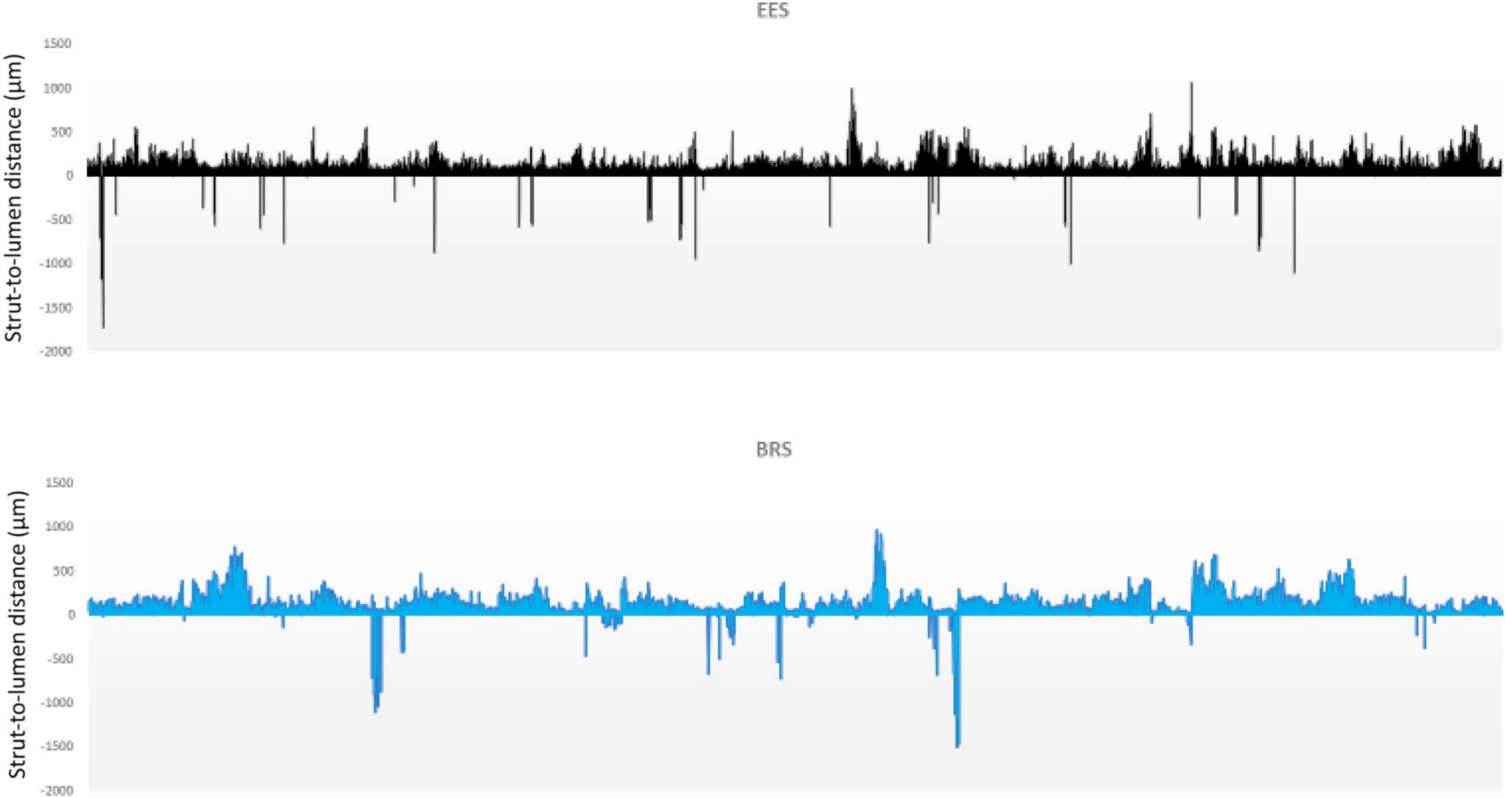
Strut-to-lumen distances plotted for all analyzed struts. Strut-to-lumen distance for each strut analyzed for all patients included in the analysis. Struts were considered covered if strut-to-lumen distance was > 20 μm for EES and > 30 μm for BRS. Negative strut-to-lumen distances indicated presence of malapposition

Strut-lumen distances obtained for all analyzed struts in both EES and BRS groups are visually represented as Fig. 5.

TLF occurred in 8 (7.8%) patients in the interval between 6 and 8 month post PCI OCT surveillance and 2 years clinical follow-up. Mean neointimal area [0.61 (0.21, 1.33) vs. 0.41 (0.11, 0.75) mm^2^, p = 0.03] and mean neointimal thickness above struts [106.1 (65.2, 214.8) vs. 80.5 (53.5, 122.1) µm, p < 0.01] were higher in patients with TLF. Patients with and without subsequent TLF had comparable GSI scores and proportions of mature ROIs (p = 0.96 and 0.39, respectively) (Data not shown).

## Discussion

The main finding of our study is that patients treated with BRS versus EES in the setting of AMI had significantly higher strut coverage as assessed by OCT at 6–8 month follow-up. Although neointimal maturity was comparable in both treatment groups in the overall cohort, in the sub-group of patients who presented with STEMI, it was significantly more advanced in BRS compared with EES. Neointimal area and thickness were both associated with subsequent TLF at 2 years post-implantation, irrespective of the implanted device. We found no association between neointimal maturity and subsequent TLF.

Apart from ISAR-Absorb MI [5], 10 other trials, namely ABSORB II [12], ABSORB III [13], ABSORB China [14], ABSORB Japan [15], AIDA [16], EVERBIO II [17], TROFI II [4], ABSORB IV [18], COMPARE-ABSORB [19] and Seo et al. [20] have compared clinical outcomes after BRS or conventional metallic EES implantation. TROFI II was the only other randomized trial to exclusively include patients presenting with myocardial infarction. Patients who underwent BVS or EES implantation in the setting of STEMI underwent protocol specified 6-month angiographic and OCT follow-up [4], making it the most apt comparator of our study. As in the present study, the proportions of uncovered and malapposed struts in TROFI II were higher in the EES group as compared to the BRS group (0.1 ± 0.4 vs. 0.0 ± 0.1%, p = 0.036) [4]. Specialized light property analysis of 6-month OCT pullbacks in the TROFI II cohort showed comparable light intensity, but lower light attenuation/ backscatter in superficial neointima in the BRS group as compared to EES [21]. This meant that BRS struts were enveloped by relatively stable superficial neointima with lower lipid components (lipid plaque, foam cells etc.) as compared to that in EES. Our GSI results are in line with the TROFI II neointimal findings, as we too report a numerically higher proportion of mature (homogeneous) neointimal ROIs in the BRS group as compared to EES, a difference that was statistically significant in our STEMI subgroup.

ABSORB BRS was officially discontinued in 2017 following concerns regarding higher rates of very late scaffold thrombosis (VLScT) compared with those observed in patients implanted with EES. Scaffold discontinuity, brought about by loss of structural integrity of BRS, which starts at around 12 months post implantation, has been identified as a leading cause of VLScT [22]. Discontinuous struts covered by thin-immature neointimal tissue comprised of fibrin or organized thrombus can dismantle and prolapse into the lumen; a process commonly known as late acquired malapposition. This exposes highly thrombogenic strut remnants to the blood, which has the potential to activate the coagulation cascade. On the other hand, mature neointimal tissue covering BRS struts should, in principle, play a critical role in long-term strut fixation. Near complete reendothelialization of BRS was observed in our studied cohort at a relatively early phase (6–8 months post-implant) as evidenced by > 97% of strut coverage with > 42% mature neointimal ROIs. This left very few struts not yet covered with critical neointimal thickness. With the majority of struts already encapsulated by neointimal tissue at 6–8 month post implant time-point, future thrombotic events caused by scaffold discontinuity in this cohort seems unlikely.

BRS have been demonstrated to be non-inferior to EES in AMI settings [4, 5]. The majority of our cohort had STEMI as presentation diagnosis (> 82%). STEMI patients tend to be younger, with proximal soft lipid-rich lesions (with little or no calcification), which are often located in large caliber vessels. Such lesions seem attractive for BRS implantation. Restoration of a normal vessel physiology after complete resorption, brought about by normalization of vasomotion and compensatory remodeling have been observed in vessels implanted with BRS [23]. Implantation of bioresorbable scaffold instead of a permanent metallic stent in principle could be an ideal strategy in STEMI patients. Uncomplicated STEMIs could thus prove to be a potential niche for future iterations of fully resorbable scaffolds.

### Limitations

Firstly, the present study involves a subset of cases from the ISAR-Absorb MI trial who underwent OCT at the time of surveillance angiography at the discretion of the operator. In such a scenario, selection-bias cannot be ruled out. Thus, some baseline characteristics between the two stent groups are slightly mismatched (EES cases were significantly older and had higher diameter stenosis at baseline). Secondly, since this was a post-hoc analysis on a subset of cases, the assessed sample size may not be enough to draw concrete conclusions, whilst still being hypothesis-generating. Thirdly, TLF assessment was done at 2 years post implant, while we know that scaffold resorption in Absorb BRS starts at around 12 months and completes at around 4 years post implantation. Irregular resorption process often leads to scaffold discontinuity which represents a primary cause of VLScT. Longer-term follow-up might be required to show a difference between the stent groups in terms of subsequent events. Fourthly, we cannot discount the fact that more aggressive lesion preparation and more frequent post-dilation used during BRS implantation caused a higher degree of vessel injury, which in turn would have contributed to the more robust healing response observed in BRS as compared to EES. This might have contributed to the observed lower rates of malapposition in patients treated with BRS. In addition, as systematic OCT imaging immediately post-procedure was not available for analysis, we are unable to comment on the evolution of stent expansion over time. Specifically, in the case of patients treated with BRS, it is possible that stent expansion reduced over time due to loss of radial strength of the scaffold, which might explain the constellation of lower rates of malapposition and lower stent expansion compared with EES.

## Conclusions

In patients who underwent OCT surveillance 6–8 months after coronary intervention for AMI, with differing implantation characteristics depending on the device type used, vessel healing was more advanced in BRS compared with EES, particularly in the STEMI subgroup. Neointimal area and neointimal tissue thickness were the only OCT parameters associated with TLF at two years post-implantation.

## Supplementary Information

Below is the link to the electronic supplementary material.

Supplementary file 1 (DOCX 34 kb)

## Abbreviations

AMI: Acute myocardial infarction
BRS: Bioresorbable-scaffold
EES: Everolimus-eluting stents
GSI: Grey-scale signal intensity
OCT: Optical coherence tomography
PCI: Percutaneous coronary intervention
QCA: Quantitative coronary angiography
ROI: Region of interest
