# Supplementary material for: Optical coherence tomography tissue coverage and characterization at six months after implantation of bioresorbable scaffolds versus conventional everolimus eluting stents in the ISAR-Absorb MI trial
Source: Int J Cardiovasc Imaging. 2021 Aug 21;37(10):2815–26. doi: 10.1007/s10554-021-02251-x (PMC8494721; doi:10.1007/s10554-021-02251-x)
Supplement: Supplementary file 1 — (DOCX 34 kb) [file 10554_2021_2251_MOESM1_ESM.docx]

**Supplementary Table 1. Baseline patient characteristics**

|  | **OCT at follow-up*** | **No OCT at follow-up*** | ***P-value*** |
| --- | --- | --- | --- |
| *Patients* | 102 | 160 |  |
| Age, yrs | 60.9±10.3 | 63.1±10.8 | 0.11 |
| Female gender | 14 (13.7) | 45 (28.1) | 0.007 |
| Diabetes mellitus | 19 (18.6) | 35 (21.9) | 0.29 |
| Insulin dependent | 2 (2.0) | 11 (6.9) | 0.07 |
| Hypertension | 44 (43.1) | 102 (63.7) | <0.001 |
| Current smoking | 54 (52.9) | 61 (38.1) | 0.05 |
| Family history of CAD | 12 (11.8) | 49 (30.6) | <0.001 |
| Prior percutaneous coronary intervention | 5 (4.9) | 17 (10.6) | 0.26 |
| Prior myocardial infarction | 4 (3.9) | 14 (8.7) | 0.21 |
| Number of vessels diseased |  |  | <0.001 |
| 1 vessel disease | 72 (70.6) | 83 (51.9) |  |
| 2 vessel disease | 19 (18.6) | 42 (26.2) |  |
| 3 vessel disease | 11 (10.8) | 35 (21.9) |  |
| Clinical presentation |  |  | 0.03 |
| ST-elevation myocardial infarction | 84 (82.4) | 113 (70.6) |  |
| Non-ST-elevation myocardial infarction | 18 (17.6) | 47 (29.4) |  |
| STEMI location/presentation |  |  | 0.95 |
| Anterior | 41 (48.8) | 55 (48.7) |  |
| Lateral | 10 (11.9) | 12 (10.6) |  |
| Posterior | 33 (39.3) | 46 (40.7) |  |
| Troponin (max), ng/dl | 5.57±8.35 | 543±3117.17 | 0.16 |

Data shown as mean±SD or number (percentage); * suitable for morphometric analysis

**Supplementary Table 2. Baseline lesion and angiographic characteristics**

|  | **OCT at follow-up*** | **No OCT at follow-up*** | ***P-value*** |
| --- | --- | --- | --- |
| *Lesions* | 102 | 160 |  |
| Target vessel |  |  | 0.78 |
| Left anterior descending | 48 (47.1) | 77 (48.1) |  |
| Left circumflex | 14 (13.7) | 26 (16.2) |  |
| Right coronary artery | 40 (39.2) | 57 (35.6) |  |
| Bifurcation | 21 (20.8) | 24 (15.0) | 0.23 |
| Pre-dilation | 86 (85.1) | 150 (94.3) | 0.01 |
| Stent diameter, max (mm) | 3.1±0.4 | 3.2±0.4 | 0.21 |
| Total stented length (mm) | 21.9±9.9 | 29.7±14.6 | <0.001 |
| Nominal diameter of largest balloon (mm) | 3.2±0.4 | 3.3±0.5 | 0.08 |
| Balloon pressure, max (atm) | 17.4±3.2 | 16.2±4.5 | 0.01 |
| Post-dilation | 40 (39.2) | 89 (55.6) | 0.009 |
| TIMI flow, post PCI |  |  | 0.36 |
| 0 | 1 (1.0) | 0 (0) |  |
| 1 | 0 (0) | 0 (0) |  |
| 2 | 2 (2.0) | 6 (3.7) |  |
| 3 | 99 (97.1) | 154 (96.2) |  |
| *Quantitative coronary angiography analysis* |  |  |  |
| Pre-intervention |  |  |  |
| Reference diameter (mm) | 2.86±0.39 | 2.95±0.45 | 0.17 |
| Minimal lumen diameter (mm) | 0.25±0.36 | 0.37±0.40 | 0.01 |
| Diameter stenosis (%) | 91.2±12.4 | 87.1±13.8 | 0.007 |
| Post-intervention |  |  |  |
| Reference diameter (mm) | 2.96±0.38 | 3.06±0.44 | 0.09 |
| Minimal lumen diameter, in-stent (mm) | 2.62±0.36 | 2.64±0.48 | 0.74 |
| Minimal lumen diameter, in-segment (mm) | 2.29±0.45 | 2.31±0.54 | 0.62 |
| Diameter stenosis, in-stent (%) | 11.2±5.8 | 13.7±9.2 | 0.005 |
| Diameter stenosis, in-segment (%) | 22.6±10.4 | 24.7±12.3 | 0.14 |

Data shown as mean±SD or number (percentage) * suitable for morphometric analysis
